# School closures due to seasonal influenza: a prospective data collection-based study of eleven influenza seasons—United States, 2011–2022

**DOI:** 10.1016/j.lana.2024.100741

**Published:** 2024-04-15

**Authors:** Nicole Zviedrite, Ferdous Jahan, Yenlik Zheteyeva, Hongjiang Gao, Amra Uzicanin

**Affiliations:** a Centers for Disease Control and Prevention, Atlanta, GA, USA; b Cherokee Nation Operational Solutions, LLC, Tulsa, OK, USA

**Keywords:** School closures, Influenza, Nonpharmaceutical interventions, Community mitigation

## Abstract

**Background:**

While numerous studies explore pandemic-associated school closures, literature is scant regarding seasonal influenza-associated closures. We previously reported summaries on COVID-19 pandemic-related school closures in the United States (US), which affected virtually all schools in the nation. The current prospective study aims to address the knowledge gap for seasonal influenza-related closures in the United States.

**Methods:**

We conducted systematic daily online searches from August 1, 2011 to June 30, 2022, to identify public announcements of unplanned school closures in the US lasting ≥1 day, selecting those that mentioned influenza and influenza-like illness (ILI) as reason for school closure (ILI-SCs). We studied ILI-SC temporal patterns and compared them with reported outpatient ILI-related healthcare visits.

**Findings:**

We documented that ILI-SCs occurred annually, with yearly totals ranging from 11 ILI-SCs in both the 2013–2014 and 2020–2021 school years to 2886 ILI-SCs in the 2019–2020 school year among more than 100,000 kindergarten through twelfth grade schools in the US. ILI-SCs occurred concurrently with widespread illness and the strongest correlations were observed during influenza A (H3N2)-dominant seasons, most notably in the 2016–2017 (Spearman rank correlation (r_s_) = 0.83) and the 2017–2018 (r_s_ = 0.84) school years. ILI-SCs were heavily centered in U.S. Department of Health and Human Services Region 4 (states of Alabama, Florida, Georgia, Kentucky, Mississippi, North Carolina, South Carolina, and Tennessee) [60% (6040/9166, Region 4/Total school closures)] and disproportionately impacted rural and lower-income communities.

**Interpretation:**

Outside of a pandemic, disease-related school closures are extreme and generally rare events for US schools and communities. Timely compilation of publicly available ILI-SC announcements could enhance influenza surveillance, particularly in severe influenza seasons or pandemics when ILI-SCs are prevalent.

**Funding:**

This work was supported by the 10.13039/100000030U.S. Centers for Disease Control and Prevention. Co-authors (NZ, YZ, HG, AU) were or are US CDC employees, and FJ was a contractor through Cherokee Nation Operational Solutions, LLC, which supported FJ’s salary, but had no additional role in the study.

**Research in context**

**Evidence before this study**

This research follows the previous publication of results based on 2-year data (school years 2011/12 and 2012/13), which showed that unplanned school closures affect millions of students annually in the US and occur for various reasons. While illness-related closures constituted only 1%, most of these were linked to respiratory illnesses and coincided with national influenza activity.

We searched PubMed for articles published between August 1, 2011 and June 30, 2022, using the keywords (“Influenza” OR “Flu” OR “Influenza-like illness”) AND (“School closure”). While numerous studies and reviews on influenza pandemic-associated school closures were identified, limited literature covers reactive closures related to seasonal influenza. Some US-based studies reported minimal impact of reactive closures, whereas studies from Japan and Russia, where closures are implemented in response to influenza-like illness (ILI) related absenteeism, suggested transmission would increase without such measures. However, nationwide studies on the geographic and temporal relationships between US ILI-related school closures (ILI-SCs) and routine influenza and ILI surveillance data were not identified.

**Added value of this study**

This study is the first in the US to describe the frequency and characteristics of school closures due to seasonal influenza/ILI over eleven consecutive seasons, establishing an inter-influenza-pandemic baseline. The data collected also offered real-time situational awareness during severe seasonal influenza outbreaks and the system was adapted to document COVID-19-associated preemptive and reactive K-12 school closures in the United States from February 2020 to June 2022.

**Implications of all the available evidence**

We observed annual occurrences of ILI-SCs, which coincided with and were likely a result of widespread illness. Publicly available ILI-SC announcements, compiled in near-real time, could be a valuable addition to existing influenza surveillance systems, especially during severe influenza seasons, pandemics when ILI-SCs are more common.

## Introduction

Because school-aged children and schools play important roles in accelerating community-wide influenza transmission, school closures implemented preemptively, i.e., before the virus is widespread, are an important nonpharmaceutical intervention reserved for severe influenza pandemics.^1^ By contrast, influenza-associated closures that occur reactively, following already heightened levels of disease, are not considered a nonpharmaceutical intervention.^1^ Despite this distinction, reactive unplanned school closures occur annually in conjunction with seasonal influenza outbreaks.^2^

We have previously reported, based on 2-year data (school years 2011–2012 and 2012–2013), that unplanned school closures (i.e., school closures not included in the academic calendar at the beginning of the school year) affect millions of students across the United States (US) annually and occur for various reasons, most frequently due to severe weather and natural disasters.^2^ While illness-related closures constituted only about 1% of all unplanned school closures, almost 60% of them were related to respiratory illnesses and their annual occurrence pattern mirrored national influenza activity.^2^

A research study conducted during the 2009 Influenza A (H1N1) pandemic documented pandemic-related school closures, where approximately 2000 schools closed reactively during the 2009 fall wave, which coincided with the fall semester of the school 2009–2010 school year.^3^ In the wake of that pandemic, and in absence of a priori assumptions, this research was initiated from 2011 in order to establish an interpandemic baseline of unplanned school closures in the US over multiple consecutive influenza transmission seasons and thereby secure the data for comparison with future seasonal and pandemic-related outbreaks.

In the present study, we describe the multi-year pattern of influenza-like illness-related school closures in the US over eleven consecutive school years, 2011–2012 through 2021–2022, with attention to geographic and temporal relationships between ILI-related school closures and routine surveillance data on medically-attended influenza and influenza-like illness (ILI) on national and regional levels. Additionally, we explore the pattern differences between the data collected before the COVID-19 pandemic and the data from the COVID-19-affected years.

## Methods

### Data collection

For this prospective data collection, from August 1, 2011, through June 30, 2022, we conducted systematic daily searches of publicly available online data (via Google and Lexis-Nexis) to identify unplanned kindergarten through twelfth grade (K–12) school closure events in the US (50 states and Washington, DC) using search strategy and data abstraction methodology described in detail by Wong et al.^2^ Closure events were documented at either the district or individual school level, based on the scope of the closure decision as reported in the announcements. For present analysis, we selected school closure events for which influenza- and/or ILI (ILI-SCs) were mentioned in the public announcements as a reason for the closure decision. School closures attributed to Coronavirus Disease 2019 (COVID-19), in absence of influenza, were excluded. From each ILI-SC announcement, we abstracted contributing factors specific to ILI and grouped them into five non-mutually exclusive categories as seen in Supplementary Table S1.

### Contextualizing influenza-related school closures

We used two separate streams of publicly available data collected by others to contextualize the data on ILI-SCs which we collected prospectively during the course of this study. One stream of these additional data included information about schools, while the other was sourced from the national influenza surveillance.

#### Data on schools

We downloaded publicly available data on school and school district characteristics from the National Center for Education Statistics’ Common Core of Data,^4^ which includes all primary and secondary public schools and districts, and Private School Universe Survey,^5^ which has reported a high coverage rate of traditional private schools (ranging from 89.6% to 98.8% from 2003–2004 to 2015–2016). Datapoints included number of schools in affected school districts, number of enrolled students, and number of staff,^4^^,^^5^ which were linked with ILI-SC data by National Center for Education Statistics school district or individual school identifiers. For school district-level ILI-SCs, we further disaggregated districts into their individual schools. National Center for Education Statistics data for the year when the ILI-SC occurred were used, when possible, or, otherwise, for the most recent year of data available. Districts with zero schools; schools with zero students; vocational, special education, and alternative schools with missing value for students; online/virtual schools; adult education programs; summer schools; jails; and schools with pre-kindergarten or transitional kindergarten as the highest grade were excluded from analysis.

#### Influenza surveillance data

We gathered publicly available surveillance data on seasonal influenza activity, including weekly national and US Department of Health and Human Services (HHS) region-specific^6^ data reporting percent of outpatient medical provider visits for influenza-like illness (ILINet) for all US states^7^ from August 1, 2011 to June 30, 2022. Definitions of HHS regions are shown in Supplementary Table S2. For the same time period, we also obtained national laboratory-confirmed influenza-associated hospitalizations of children and adults data (FluSurv-NET) reported by thirteen US states (California, Colorado, Connecticut, Georgia, Maryland [Baltimore metropolitan area], Michigan, Minnesota, New Mexico, New York, Ohio, Oregon, Tennessee, and Utah).^8^ Additionally, we collected information on reported seasonal influenza severity and predominant strain(s).^9^

### Data management and analysis

Data were imported into SAS 9.4 (SAS Institute Inc., Cary, North Carolina) for analysis. Descriptive statistics were calculated to summarize characteristics of ILI-SCs (cause, seasonality, duration, and geographic distribution) and impacts on closed schools (number of students and teachers affected).

We studied temporal patterns of weekly occurrence of ILI-SCs and compared them with seasonal influenza surveillance data. We calculated Spearman rank correlations (r_s_) to evaluate these relationships (at alpha = 0.05) during influenza seasons (defined as epidemiological weeks 40 through 20 (ILINet), or through week 17 (FluSurv-NET)). We first assessed the monotonicity assumption for Spearman’s rank correlation by scatterplots between the number of ILI-SCs and weekly surveillance data. p-value calculation for the Spearman’s rank correlation was based on Fisher’s Z-transformation. ILINET data are available for all US states, and we used them for calculating correlations between the ILI-SC data and the medically-attended ILI outpatient visits. In contrast, influenza hospitalization data in FluSurv-NET are collected in only thirteen states and not nationwide.^8^ Therefore, we limited exploring the correlations between ILI-SCs and FluSurv-NET data to only these thirteen states reporting influenza hospitalization data. The final week of each calendar year (epidemiological weeks 52 (2011–2013, 2015–2019, 2021) or 53 (2014, 2020)) was excluded from analysis based on the assumption that all schools in the nation are on winter break during that period. In the 2019–2020 season, we truncated the season after epidemiological week 11 of 2020 (March 8–14, 2020), which was the last week an ILI-SC was captured by our searches. Despite this, data from January and February 2020 may have been impacted by unrecognized transmission of severe acute respiratory syndrome coronavirus 2 (SARS-CoV-2), the causative agent of COVID-19. Truncation coincides with nationwide school closures implemented by states in response to the known spread of SARS-CoV-2, whereby approximately 100,000 public schools closed in March 2020.^10^

This project underwent ethical review at the Centers for Disease Control and Prevention’s Human Research Protections Office and was determined not to involve human subjects; it was therefore not subject to institutional review board review requirements.

This study followed the STROBE guidelines.^11^

Supplementary Methods for additional analyses are described in Supplementary material.

### Role of the funding source

This work was supported by the Centers for Disease Control and Prevention. The co-authors are or were employees (NZ YZ HG AU) or contractors (FJ) of the US CDC at the time of the study. Ferdous Jahan (FJ) was employed by Cherokee Nation Operational Solutions, LLC. The funder (Cherokee Nation Operational Solutions, LLC) provided support in the form of salary for the author (FJ) but did not have any additional role in the study design, data collection and analysis, decision to publish, or preparation of the manuscript. The specific roles of all authors are articulated in the ‘Author Contributions’ section.

## Results

From August 1, 2011 to June 30, 2022, we found 2077 school closure events attributed to influenza/ILI; the average number of ILI-SC events per year during the pre-COVID-19 period (2011–2012 to 2019–2020) was 224.3 (median 112), while the average for the COVID-19-affected period (2020–2021 and 2021–2022) was 29 (5 and 53 events, respectively) (Table 1). In both the pre-COVID-19 and COVID-19-affected periods, the most frequently specified reason for closure was increased absenteeism of students and staff due to illness [1358 of 2019 (67.3%) and 34 of 58 (58.6%), respectively] (Table 2).

**Table 1:** Publicly announced school closures associated with influenza-like illness, by school year—United States, August 1, 2011–June 30, 2022.

School year(s)	Number of school closure events^a^^,^^g^, n (column %)	Estimated number of schools closed^b^^,^^g^			Estimated number of students affected^d^, n	Estimated number of teachers affected^d^^,^^e^, n
		By school closure type, n (row %)		Total, n (column %)		
		District-wide^c^	Individual school			
2011/2012	33 (2)	87 (84)	16 (16)	103 (1)	38,167	2555
2012/2013	104 (5)	352 (92)	31 (8)	383 (4)	142,223	9091
2013/2014	5 (0)	8 (73)	3 (27)	11 (0)	2659	170
2014/2015	112 (5)	249 (81)	59 (19)	308 (3)	123,506	8007
2015/2016	20 (1)	30 (75)	10 (25)	40 (0)	13,553	853
2016/2017	231 (11)	1237 (96)	55 (4)	1292 (14)	612,176	39,700
2017/2018	458 (22)	1820 (93)	146 (7)	1966 (22)	901,357	58,026
2018/2019	429 (21)	1775 (93)	137 (7)	1912 (21)	890,750	57,677
2019/2020	627 (30)	2736 (95)	150 (5)	2886 (32)	1,289,644	82,812
2020/2021	5 (0)	8 (73)	3 (27)	11 (0)	5902	437
2021/2022	53 (3)	215 (96)	9 (4)	224 (2)	100,924	6698
Pre-COVID-19 years^f^: 2011/2012 to 2019/2020	2019 (97)	8294 (93)	607 (7)	8901 (97)	4,014,035	258,891
COVID-19-affected years: 2020/2021 to 2021/2022	58 (3)	223 (95)	12 (5)	235 (3)	106,826	7135
Total	2077	8517 (93)	619 (7)	9136	4,120,861	266,026

a Closure events were documented at either the district-level or the individual school level, based on the source and scope of the closure decision as reported in the announcements.

b Schools were counted once for each time they were part of a school closure event at either the district-level or school level.

c Numbers of schools in district-level closure events were estimated based on the number of kindergarten through twelfth grade schools in each affected school district per data available from the National Center for Education Statistics.^3^

d Students and teachers were counted once for each school closure event.

e Part-time teaching positions were reported as a fraction of one full-time position.

f The 2019-2020 school year is included in pre-COVID-19 seasons and includes influenza- and influenza-like illness-associated school closures through epidemiological week 11 of 2020, the last week an ILI-related closure was reported in the school year. While the first COVID-19-related school closure occurred during epidemiological week 9 in 2020, widespread COVID-19-related school closures were reported during the subsequent epidemiological weeks 12 and 13 of 2020.^10^

g Percentages may not add up to 100%, as they are rounded to the nearest percent.

**Table 2:** Reasons^a^ for influenza-like illness-related school closure events, by school year—United States, August 1, 2011–June 30, 2022.

	School years													Total^f^
	Pre-COVID-19 Years^f^										COVID-19-affected Years^f^			
	2011/2012	2012/2013	2013/2014	2014/2015	2015/2016	2016/2017	2017/2018	2018/2019	2019/2020^b^	Sub-total	2020/2021	2021/2022	Sub-total	
Number of school closure events, no. (%)	33 (2)	104 (5)	5 (0)	112 (5)	20 (1)	231 (11)	458 (22)	429 (21)	627 (30)	2019	5 (0)	53 (3)	58	2077
Reasons for school closure decision as stated in announcements of ILI-SCs, no. (%)														
Announcement mentions only ILI/illness^c^	6 (18)	40 (38)	0	30 (27)	3 (15)	86 (37)	111 (24)	82 (19)	115 (18)	473 (23)	0	9 (17)	9 (16)	482 (23)
Announcement mentions ILI + other factors^d^	27 (82)	64 (62)	5 (100)	82 (73)	17 (85)	145 (63)	347 (76)	347 (81)	512 (82)	1546 (77)	5 (100)	44 (83)	49 (84)	1595 (77)
Other factors mentioned in the closure announcements, related to ILI, no. (%)														
Student and staff absenteeism^e^	21 (64)	54 (52)	4 (80)	67 (60)	15 (75)	125 (54)	280 (61)	327 (76)	465 (74)	1358 (67)	3 (60)	31 (58)	34 (59)	1392 (67)
To stop/halt/prevent illness spread	7 (21)	14 (13)	1 (20)	15 (13)	3 (15)	24 (10)	77 (17)	49 (11)	73 (12)	263 (13)	0	4 (8)	4 (7)	267 (13)
To clean/disinfect classrooms, buildings, and facilities	4 (12)	4 (4)	0	9 (8)	0	21 (9)	85 (19)	43 (10)	86 (14)	252 (12)	2 (40)	9 (17)	11 (19)	263 (13)
To allow students/teachers to rest and recover	4 (12)	6 (6)	0	5 (4)	1 (5)	23 (10)	55 (12)	63 (15)	62 (10)	219 (11)	0	2 (4)	2 (3)	221 (11)
Due to financial reasons	1 (3)	1 (1)	0	0	1 (5)	1 (0)	0	1 (0)	0	5 (0)	0	0	0	5 (0)

a Reasons are recorded as stated in the school closure announcement.

b The 2019-2020 school year is included in pre-COVID-19 seasons and influenza- and influenza-like illness-associated school closures (ILI-SCs) through epidemiological week 11 of 2020, the last week of an ILI-related closure in the school year. The first COVID-19-related school closure occurred during epidemiological week 9 in 2020, however widespread COVID-19-related school closures were reported during the subsequent epidemiological weeks 12 and 13 of 2020.^10^

c School closure announcements in general terms attribute closure to influenza or influenza-like illness (ILI), or ILI and other illnesses or causes, and do not specify any other ILI-related factors.

d School closure announcements attribute closure to ILI (or ILI and other illnesses or causes) and specify one or more of the additional ILI-related factors. Categories are not mutually exclusive because a closure announcement may attribute the closure to more than one factor and/or there may be more than one announcement, which attribute the closure to different factors.

e Includes statements which indicate a closure due to increased illness or absenteeism among students and/or staff.

f Percentages may not add up to 100%, as they are rounded to the nearest percent.

**Fig. 1:**
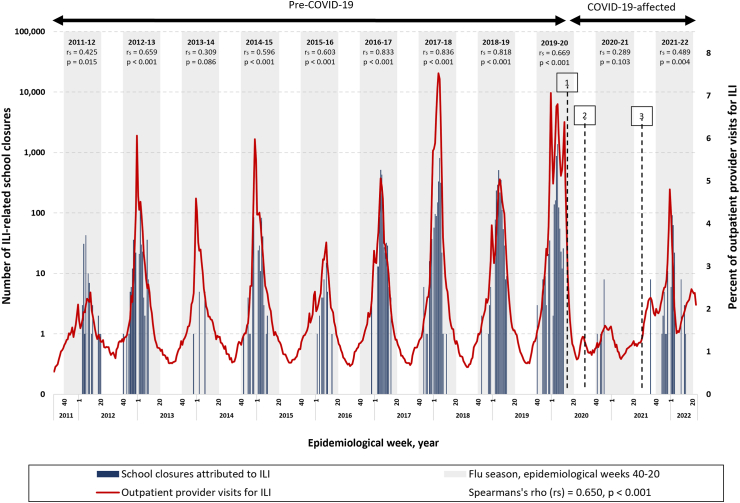
**Estimated number of school closures associated with influenza-like illness (ILI) (N = 9136) and percent of outpatient provider visits for ILI∗ by epidemiologic week—United States, August 1, 2011—June 30, 2022.** ∗Percent of outpatient provider visits for ILI per data available from ILINet.^6^ 1) Schools closed nationwide due to spread of SARS COV2.^10^ 2) The majority of the schools opened virtually for the 2020–2021 SY, with in-person learning increasing over the school year.^12^ 3) Schools reopened for the 2021–2022 SY with the majority in person.^13^ Blue bars represent school closures attributed to influenza and influenza-like illness (ILI) and refer to the left Y-axis. The red line represents the percent of outpatient provider visits for ILI and refer to the right y-axis. Note: In the 2012–13, 2014–15, 2016–17, and 2017–18 influenza seasons, influenza A (H3N2) was the predominant strain.^8^ In the 2011–12 influenza season, which was unusually mild, influenza A (H3N2) predominated overall but, influenza A (H1N1)pdm09 and influenza B also widely circulated.^8^ In the 2013–14 and 2015-16 seasons, influenza A (H1N1)pdm09 was the predominant strain.^8^ In the 2018-19 season, there were two peaks of similar magnitude dominated by influenza A (H1N1) followed by influenza A (H3N2).^8^ During the 2019–2020 season, Influenza B predominated early in the season followed by influenza A (H1N1)pdm09.^8^ In the 2020–2021 season, there was unusual low flu activity in the United States when both Influenza A ((H1N1)pdm09)and (H3N2)) and influenza B, and the majority of influenza A viruses were H3N2.^8^ In the 2021–2022 season, the majority of positive flu tests reported to the CDC by US Public Health Laboratories were attributed to influenza A (H3N2).^8^

Over the 11-year study period, the 2077 ILI-SC events accounted for an estimated 9136 school closures and affected an estimated 4.1 million students and 260,000 teachers (Table 1, Fig. 1). Nearly two-thirds of all ILI-SCs [6040 of 9136 (66.1%)] occurred in HHS Region 4 (states of Alabama, Florida, Georgia, Kentucky, Mississippi, North Carolina, South Carolina, and Tennessee), one of two regions to have at least one ILI-SC announced in every year of the study, the other being HHS Region 5 (states of Illinois, Indiana, Michigan, Minnesota, Ohio, and Wisconsin) which accounted for one-tenth of ILI-SCs [991 (10.8%)] (Supplementary Table S3). By contrast, ILI-SCs were seldom reported in some of the other regions. For example, in Region 1 (states of Connecticut, Maine, Massachusetts, New Hampshire, Rhode Island, and Vermont), twenty-one ILI-SCs were identified and in Region 9 (states of Arizona, California, Hawaii, and Nevada) only fourteen ILI-SCs were identified during the study period (Supplementary Fig. S2 and Table S3). Correlation between ILI-SCs and ILINet data varied by HHS Region. Across eleven school years, moderate correlation between ILI-SCs and outpatient ILI surveillance was observed in Region 4 [r_s_ = 0.66 (p < 0.001)], Region 5 [r_s_ = 0.61 (p < 0.001), and Region 6 (states of Arkansas, Louisiana, New Mexico, Oklahoma, and Texas) [r_s_ = 0.75 (p < 0.001)] (Supplementary Fig. S2 and Table S4). In other regions the correlation was weak or very weak.

### Pre-COVID-19 school years (2011–2012 to 2019–2020)

Over the initial nine years of the study period, encompassing the 2011–2012 to 2019–2020 school years, 2019 observed ILI-SC events accounted for an estimated total of 8901 school closures (Table 1). The median duration of closure was 2 school days (range: 1–7) (Supplementary Table S3). The largest number of ILI-SCs [2886 of 8901 (32.4%)] were captured in the 2019–2020 school year prior to the onset of the COVID-19 pandemic in March 2020 (Table 1). The prior two school years, 2017–2018 and 2018–2019, also had high numbers with 1966 (22.1%) and 1912 (21.5%) ILI-SCs, respectively. Additional details regarding repeat school closures within and across school years can be found in Supplementary Tables S7–S9.

During the pre-COVID-19 period, schools located in rural areas had significantly higher odds of experiencing ILI-SCs compared with schools in cities (aOR 8.78, 95% CI 7.93–9.72), followed by towns (town vs city, aOR 6.96, 95% CI 6.25–7.76) (Supplementary Table S10). Schools with a higher percentage of students enrolled in the federal free or reduced-price lunch program had significant odds to close schools (with 10% increase of enrollment, aOR 1.15, 95% CI 1.14–1.17).

The timing of ILI-SC occurrence on the national level in relation to outpatient ILI activity varied across influenza seasons (Fig. 1). The time series begins with the 2011–2012 season, the mildest influenza season observed, which only reached the national baseline of percent of outpatient provider visits for ILI, as reported by ILINet, for a period of one week and all closures occurred after winter break (Supplementary Fig. S3). In the following three seasons, ILI activity breached the national baseline in late fall and peaked near the first of the year, while the peaks and majority of ILI-SCs followed a few weeks after. In the subsequent four seasons, relatively later peaks (in February and March) of ILI activity were preceded or met by the majority of ILI-SCs. During the 2019–2020 season, ILI activity surpassed the national baseline in early November and was characterized by three peaks; the second of these, in early February, coincided with the peak of ILI-SCs. As school closed preemptively and nearly synchronously nationwide as a countermeasure to the start of the COVID-19 pandemic,^10^ ILI activity sharply declined from early March; no additional ILI-related school closures were observed beyond March 11, 2020 (Fig. 1, Supplementary Fig. S3).

The strongest correlations (>r_s_ = 0.80) between ILI-SCs and national outpatient healthcare visits for ILI occurred in the three seasons between 2016-2017 and 2018–2019, which were dominated, all or in-part, by influenza A (H3N2) (Supplementary Table S4). Strong correlation was seen in the 2019–2020 season, dominated in turn by both influenza A (H1N1) and influenza B, when truncated to the last observed ILI-SC in epidemiological week 11 (week of March 8, 2020) [r_s_ = 0.77 (p < 0.001)]. Truncation at the preceding and following weeks led to lower correlations.

**Fig. 2:**
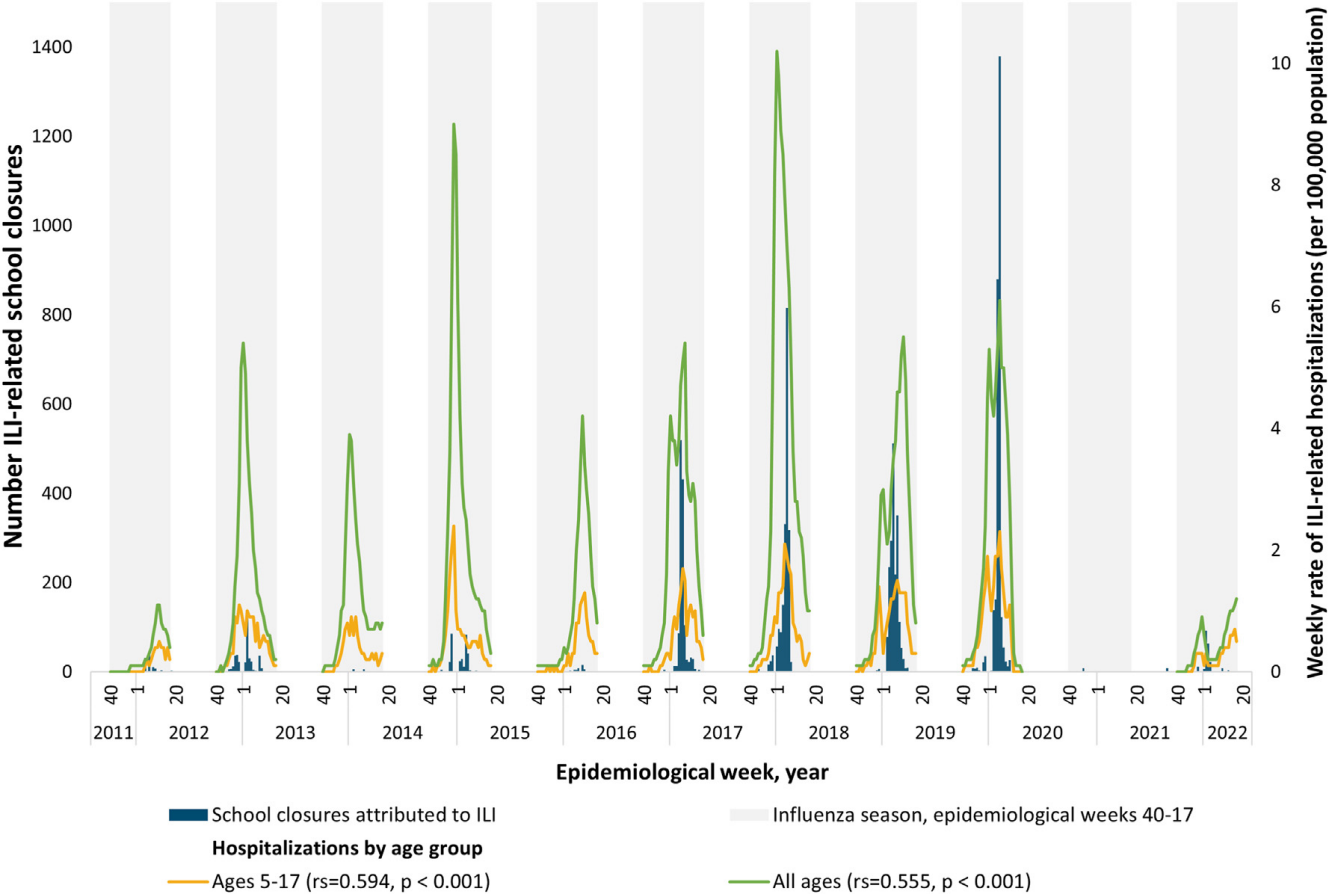
**Estimated number of school closures associated with influenza-like illness and laboratory-confirmed influenza-associated hospitalizations∗—13 states**^**†**^**, August 1, 2011—June 30, 2022.** ∗Data on laboratory-confirmed influenza-associated hospitalizations per data from FluSurv-NET^7^ are shown for two groups, “all ages” and “ages 5–17”. Ages 5–17 reflect school-age children, which had the strongest correlation with influenza/ILI-related school closures. Lines for hospitalization data are not continuous because data are only reported for epidemiological weeks 40-17. ^†^Data on laboratory-confirmed influenza-associated hospitalizations^7^ were available from the following states: CA, CO, CT, GA, MD, MN, NM, NY, OR, TN, MI, OH, and UT. Blue bars represent school closures attributed to influenza and influenza-like illness (ILI) and refer to the left Y-axis. The yellow line represents the rate of influenza-related hospitalizations for ages 5–17 years and the green line represents the rate of influenza-related hospitalizations for all age groups, both refer to the right y-axis. Note: In the 2012–2013, 2014–2015, 2016–2017, and 2017–2018 influenza seasons, influenza A (H3N2) was the predominant strain.^8^ In the 2011–2012 influenza season, which was unusually mild, influenza A (H3N2) predominated overall but, influenza A (H1N1)pdm09 and influenza B also widely circulated.^8^ In the 2013–2014 and 2015–2016 seasons, influenza A (H1N1)pdm09 was the predominant strain.^8^ In the 2018–2019 season, there were two peaks of similar magnitude dominated by influenza A (H1N1) followed by influenza A (H3N2).^8^ During the 2019–2020 season, Influenza B predominated early in the season followed by influenza A (H1N1)pdm09.^8^ In the 2020–2021 season, there was unusual low flu activity in the United States when both Influenza A ((H1N1)pdm09)and (H3N2)) and influenza B, and the majority of influenza A viruses were H3N2.^8^ In the 2021–2022 season, the majority of positive flu tests reported to the CDC by US Public Health Laboratories were attributed to influenza A (H3N2).^8^ Note: Spearman rank correlations were used to evaluate the relationship between Influenza/ILI-related school closures and laboratory-confirmed influenza-associated hospitalizations during influenza seasons (epidemiological weeks 40 through 17).

A moderate correlation was noted between the ILI-SCs and all-age laboratory-confirmed influenza-associated hospitalizations in the 13 states that routinely report these data [r_s_ = 0.56 (p < 0.001); Fig. 2, Supplementary Table S5]. Correlation was slightly higher for those in the 5–17 years age group, which aligns with K-12 students, [r_s_ = 0.59 (p < 0.001)] [Supplementary Table S5].

### COVID-19-affected school years (2020/2021 to 2021/2022)

In the two complete school years following the onset of the COVID-19 pandemic, 2020–2021 and 2021–2022, 235 schools closed as part of 58 ILI-SC events (Table 1). All ILI-SCs in 2020–2021 (n = 11) and 200 of 224 (89.3%) in 2021–2022 were attributed to both ILI and COVID-19 (Supplementary Table S6). ILI-SCs in the 2020–2021 school year were the longest in the study period, with a median of 4 days (range 3–33) as compared to medians of 1–2 days in all other years including 2021–2022 (Supplementary Table S3).

In the 2020–2021 season, ILI activity did not surpass the national baseline and all ILI-SCs preceded the winter holidays (Supplementary Fig. S3). In the 2021–2022 season, ILI activity breached the national baseline but for fewer weeks as compared to all pre-COVID-19 seasons except 2011–2012, and ILI-SCs reached their height a couple weeks after the ILI peak. The weakest correlation between annual ILI-SCs and national outpatient healthcare visits was observed in 2020–2021 [0.29 (p = 0.103)] (Supplementary Table S4). Likewise, correlation between ILI-SCs and laboratory-confirmed influenza-associated hospitalizations were weak for all ages [0.32 (p = 0.011)] and not significant for the 5–17 age group during the COVID-19-affected period [0.22 (p = 0.087)] (Supplementary Fig. S4 and Table S5).

## Discussion

Our data demonstrate that ILI-SCs occur annually in the US with temporality that reflects the general patterns of influenza activity on both national and regional levels as observed through routine surveillance of medically attended ILI. In most seasons prior to the COVID-19 pandemic, school winter breaks temporarily reduced ILI activity with seasonal peaks usually following within a few weeks of the start of second semester. ILI-SC annual peaks occurred in the same timeframe, presumably once the number of ill students reached a critical mass a few weeks after school re-opening for spring semester. A similar pattern was exhibited around the winter break of the 2019–2020 school year; however, the advent of the COVID-19 pandemic and subsequent preemptive closure of nearly all K-12 schools in the US likely impacted the remaining weeks of both the season’s ILI activity and related SCs. During the subsequent two COVID-affected years, 2020–2021 and 2021–2022, ILI-SCs reflected the temporality and low levels of ILI activity.

Prior to the COVID-19 pandemic, the highest numbers of ILI-SCs occurred in seasons when influenza A (H3N2) was predominant either nationally or regionally. Among these, the 2017–2018 and 2018–2019 seasons each reached a level of closures previously only seen in the 2009 pandemic where a reported 1947 schools closed.^3^ Meanwhile, exceptionally mild seasons, such as 2011–2012 or those dominated by influenza A (H1N1) generally had far fewer closures.

However, the largest annual number of ILI-SCs during the study period occurred during the 2019–2020 school year, which was affected by an intense seasonal outbreak predominated first by influenza B and subsequently by influenza A (H1N1). This record was set despite the school year being abruptly interrupted, by the implementation of measures used to mitigate the spread of SARS-CoV-2 as early as February 2020 and ultimately by the near simultaneous preemptive closure of all K-12 schools beginning in the week ending March 14, 2020 in order to slow transmission of SARS-CoV-2.^10^^,^^14^

In addition to variations in seasonal influenza epidemiology, the number of ILI-SCs per year may have been influenced by differences in seasonal vaccine effectiveness. The higher counts of ILI-SCs in H3N2-dominated years may, at least in part, be related to a relatively higher effectiveness of the influenza A (H1N1) vaccine component compared with the A (H3N2) component.^15^ Furthermore, because the predominant circulating virus types and subtypes can vary geographically,^7^ vaccine effectiveness may also differ by locale.

Though vaccine effectiveness may be a contributing factor to seasonal distribution of ILI-SC occurrence, uneven geographic distribution could also have been related to variations in vaccine uptake or certain programmatic factors that prompt school authorities to close schools, which may be common to a state or locale.

There were observable regional differences in vaccine coverage across the study period. Among HHS Regions with relatively few ILI-SCs across the study period, particularly regions 1, 2, 3, 7, and 8 (see Supplementary Fig. S1 and Table S2 for subordinated states), estimated vaccine coverage across the study period tended to be higher than national estimates. Meanwhile HHS Region 4, the region with the most ILI-SCs, reported vaccine coverage estimates consistently below the national-level.^16^ This may suggest that seasonal vaccine uptake is a contributing factor in ILI-SC occurrence, and, while not a key driver, potentially has a bigger impact in some years than others.

There are likely also programmatic factors that impact school closure decisions. For example, regional variability could be affected by differences in state policies related to school funding or the varying costs to run schools. Student enrollment is typically a primary driver of funds provided to schools by the state, and, depending on how the number of enrolled students is calculated in each state, school closure decisions may be swayed by illness-related absenteeism given the connection between attendance and funding.^17^ Additionally, factors such as the higher costs of food service and transportation in rural schools^18^ may impact the cost-effectiveness of running differently located schools with high levels of illness-related absenteeism. More research is needed to analyze the effect of policies and programmatic factors on the reasons for and barriers to closure. Disproportionate burden of ILI-SCs appears to be concentrated in socio-economically disadvantaged communities of two HHS regions, which adds urgency to further evaluate and, where needed, address programmatic factors that may be influencing these trends among already marginalized and underserved populations.

During the COVID-dominated period (school years 2020–2021 and 2021–2022), ILI-SC data collection continued but remarkably few ILI-SCs were documented. The 2020–2021 school year had among the lowest number of ILI-SCs, comparable only to the 2013–2014 school year, a moderate influenza A (H1N1)-dominated season. Moreover, all documented ILI-SCs in the 2020–2021 school year were simultaneously attributed to COVID-19, and the weight of each disease’s contribution to the closure decisions is unknown. The low number of observed ILI-SCs in the 2020–2021 school year is credible, because the ILI activity that year did not breach the national baseline, a first during the study period. While the 2021–2022 school year saw an increase in ILI-SCs from the previous year, the numbers were only a fraction of the number of ILI-SCs in the three school years prior to the COVID-19 pandemic, all dominated by influenza A (H3N2). This pattern is consistent with the generally low ILI activity observed during the COVID-dominated years.^14^

In response to the growing levels of community SARS-CoV-2 transmission in the summer and fall of 2020, many schools and districts across the country offered a variety of reduced-contact learning options in the 2020–2021 school year including hybrid (partly in-person/partly distance learning) and fully virtual (online) education models, thereby limiting the density, frequency, and duration of in-person student congregation in schools.^12^ The nearly total return to in-person learning in the 2021–2022 school year^19^ coincided with an uptick in both ILI-activity and ILI-SCs.

ILI-SCs described in this multi-year study are reactive closures associated with increased ILI activity, i.e., they occurred as a consequence of increased disease transmission as reported in most school closure announcements. In contrast, during the 2009 Influenza A (H1N1) pandemic, both preemptive and reactive closures were implemented in the US and other countries.^13^ Subsequently performed systematic literature reviews support pre-emptive school closures as a nonpharmaceutical intervention to reduce virus spread in schools and surrounding communities,^20^^,^^21^ but the effectiveness of closures implemented reactively remains unclear. Two US-based studies reported that reactive closures have little effect during seasonal^22^ or pandemic^23^ influenza outbreaks; conversely, studies implemented elsewhere indicate that transmission would be even greater if they are not implemented,^24^^,^^25^ though some with only minor effect.^25^ Reactive closures have for decades been routinely used in Japan and Russia to control outbreaks of seasonal and pandemic influenza where they are triggered locally by defined levels of heightened ILI-related student absenteeism.^24^^,^^26^ However, it has yet to be shown under what conditions reactive closures might help control local outbreaks in the US. Meanwhile, recent US-based research has shown that routine monitoring of cause-specific student absenteeism can act as an early warning signal of increased ILI activity in schools and surrounding community.^27^ For communities which are disproportionally burdened year-after-year with ILI-SCs, it may be particularly worthwhile to explore more effective alternatives to late reactive ILI-SCs, even if only via better (earlier) timing of the interruptions of in-person instruction to obtain greater reduction of within-school disease transmission.

This evaluation is subject to at least four limitations. First, ILI-SC data only captured closures reported through publicly available online media sources, potentially missing announcements made exclusively through other communication means (e.g., text messages, listservs, etc.). While we took care to crosscheck public announcements of closure with publicly available school and school district sources (e.g., school and school district websites and social media pages), there was no internal process of validation of the data we compiled via these systematic daily searches of public announcements, as this would have been far beyond the scope and scale of this study and the resources available for this work. However, we previously shared portions of our data with outside researchers whose subsequent work provides cross-validation with other sources of publicly available data on school closures (ex., Twitter, Facebook).^28^ Second, because data points for ILI-SCs were abstracted exclusively from publicly available announcements, some information may not have been complete or entirely accurate. However, the method of data collection imposed no reporting burden on schools and was relatively inexpensive. Third, data on outpatient healthcare provider visits for ILI and laboratory-confirmed influenza hospitalizations were subject to limitations that are characteristic of surveillance systems. Additionally, not all states were included in hospitalization data and therefore results are not generalizable. However, this was mitigated by the fact that Tennessee, the state most greatly impacted by ILI-SCS, was accounted for in the hospitalization data. Lastly, we compared the relationships of ILI-SCs and ILI surveillance data on national and sub-national levels. Such high level of comparisons may not have fully reflected relationships on the local level (i.e., communities around closed schools), and further studies exploring these relationships on the local level are warranted.

This study describes the frequency and characteristics of US school closures attributed to seasonal influenza/ILI over eleven consecutive influenza seasons, and thereby addresses an important knowledge gap for the United States by establishing an inter-influenza-pandemic baseline of such closures. This data collection also provided real-time situational awareness information during severe seasonal influenza outbreaks and, after suitable modification, was instrumental in documenting preemptive and reactive COVID-19-associated K-12 school closures in the US from March to June 2020,^10^ and reactive COVID-19-associated school closures in the US in the 2020–2021 (n = 16,890) and 2021–2022 (n = 19,871) school years.^29^ The ILI-SC data, systematically collected from public sources, have been congruent with trends observed via national disease surveillance systems both during high ILI activity seasons as well as during the unusually low ILI incidence in COVID-affected years. Of note, during the study implementation ILI-SC data were available about a week sooner than ILINet data, i.e., ILI-SC data were being compiled in near-real time.

### Conclusion

Given that ILI-SC announcements are publicly available and, as demonstrated in course of this research, amenable to compiling in near-real time, the routine monitoring of ILI-SCs may be a useful addition to existing influenza surveillance systems to detect impact of influenza on schools, particularly during severe influenza seasons or pandemics when ILI-SCs are likely to occur more frequently. To that end, we believe validation would be an appropriate step if this research project were to be continued as a formalized surveillance system. Furthermore, novel tools that were not available during this study are currently emerging and may make such undertakings even more doable (e.g., AI-based solutions for natural language processing). An alternative approach would be to initiate direct data collection from schools via electronic reporting of disease-related or indeed all unplanned school closures lasting ≥1 day to state education agencies and organize data sharing at the federal level, but that would likely require a significantly greater effort. The reported economic burden of ILI-SCs documented by this study in the pre-COVID period is disproportionally concentrated in socio-economically disadvantaged parts of two HHS regions,^30^ which further underscores the importance of continued ILI-SC monitoring and research as we prioritize addressing health disparities and impacts on marginalized communities. Meanwhile, given that ILI-SCs appear to be a drastic measure with little public health benefit for affected communities because they are undertaken by school authorities reactively, i.e., when within-school ILI activity has already reached critical proportions resulting in high student absenteeism, ways to reduce ILI disease burden in schools merit continued exploration. A multi-pronged approach that includes increased student and staff vaccination, better ventilation, and timely implementation of nonpharmaceutical interventions in combination with the monitoring of cause-specific absenteeism, could provide opportunities to timely reduce ILI transmission in schools before high student absenteeism escalates into a late reactive school closure.

## Contributors

AU, HG, and YZ conceptualized the data collection and then developed the original methodology, with later contributions by NZ and FJ. NZ and FJ conducted data collection, abstraction, and validation with support from staff noted in the acknowledgments. FJ and NZ conducted the formal analysis with statistical support from HG, and developed visualizations. An early draft was written by YZ using a shorter time series and NZ was responsible for the draft regarding the full timeseries. All authors contributed to the review and editing of the manuscript, with AU also providing project administration and supervision. AU has responsibility for the decision to submit the study for publication.

## Data sharing statement

Data informing the preceding analyses were collected from publicly available data sources, as described in the manuscript. Search terms, search parameters, and data abstraction methods are described in detail in the manuscript to enable replication of this work. Furthermore, data will be made publicly available at data.cdc.gov in summer 2024. Inquiries related to these data should be directed to Nicole Zviedrite (nzviedrite@cdc.gov).

## Declaration of interests

Ferdous Jahan (FJ) was employed by Cherokee Nation Operational Solutions, LLC. The funder (Cherokee Nation Operational Solutions, LLC) provided support in the form of salary for the author (FJ) but did not have any additional role in the study design, data collection and analysis, decision to publish, or preparation of the manuscript. All other authors declare no interests.
